# A rabbit anti-human CD38 antibody for eliminating daratumumab and isatuximab interference in immunohematology testing

**DOI:** 10.3389/fimmu.2026.1726341

**Published:** 2026-02-10

**Authors:** Liangzi Zhang, Yinze Zhang, Jun Qi, Xiaomin Su, Qinqin Zuo, Fang Yan, Ying Cui, Na Li, Yanying Dong, Hua Xu

**Affiliations:** 1 Shaanxi Blood Center, Xi’an, Shaanxi, China; 2 Department of Blood Transfusion, Shenzhen University General Hospital, Shenzhen, China; 3 Department of Blood Transfusion, The Affiliated Hospital of Northwest University, Xi’an No.3 Hospital, Xi’an, Shaanxi, China; 4 Department of Blood Transfusion, The First Affiliated Hospital of Xi’an Jiaotong University, Xi’an, Shaanxi, China; 5 Department of Blood Transfusion, Tangdu Hospital, The Fourth Military Medical University, Xi’an, Shaanxi, China; 6 Department of Transfusion Medicine, The Second Affiliated Hospital of Xi’an Jiaotong University, Xi’an, Shaanxi, China

**Keywords:** anti-CD38 antibodies, antigen masking indirect antiglobulin test, blood transfusion, rabbit monoclonal antibody, serological testing

## Abstract

Anti-CD38 antibodies such as daratumumab (DARA) and isatuximab (ISA), used in multiple myeloma (MM) therapy, bind to CD38 on red blood cells (RBCs), leading to pan-agglutination in indirect antiglobulin tests (IATs) and interfering with pretransfusion testing. Given the frequent need for transfusions in MM patients with anemia, resolving this interference is clinically essential. While dithiothreitol (DTT) is commonly used, it compromises clinically important RBC antigens. To address this limitation, a novel approach was developed using a recombinant rabbit anti-human CD38 IgG monoclonal antibody, D2, to treat RBCs. Rabbits were immunized with hCD38-His. Antigen-specific B cells were isolated via flow cytometry and subsequently sequenced to obtain the antibody genes. After cloning and expression, a rabbit mAb named D2 was selected for its ability to bind the CD38 on human RBCs and block the binding of therapeutic anti-CD38 antibodies without affecting other RBC blood group antigens. Incubation of 1 μL of packed RBCs with 3 μL of 1 mg/mL D2 for 10 minutes at room temperature eliminated DARA- or ISA-induced pan-agglutination in the IAT while preserving the detection of clinically significant irregular antibodies. Its efficacy was confirmed using samples from 48 DARA- or ISA-treated patients. D2 is stable, easy to use, and suitable for routine application in transfusion laboratories to resolve anti-CD38-induced interference in pretransfusion testing.

## Graphical Abstract

**Figure:**
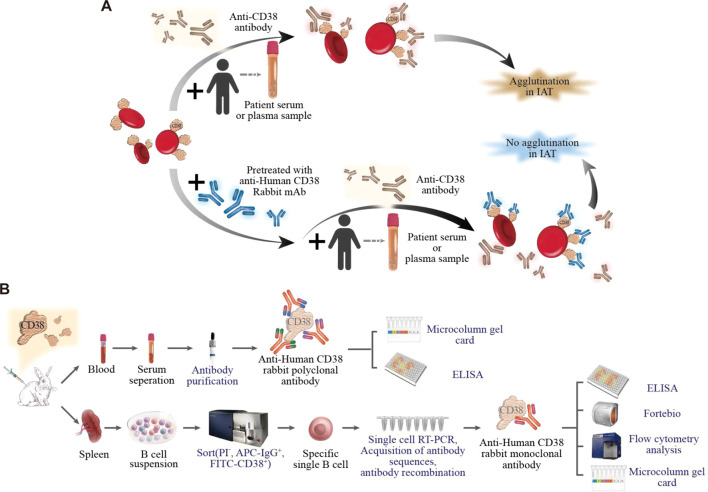
CD38 masking mechanism and antibody generation workflow. **(A)** Mechanism by which rabbit anti-human CD38 monoclonal antibody masks CD38 on red blood cells, preventing therapeutic antibody binding and allowing detection of irregular antibodies. **(B)** Workflow for generation of rabbit antihuman CD38 polyclonal and monoclonal antibodies.

## Introduction

Monoclonal antibodies (mAbs) have transformed therapy owing to their specificity, long half-life, and favorable safety profile. Anti-CD38 mAbs, such as daratumumab (DARA) (1–3) and isatuximab (ISA) (4), are effective in multiple myeloma (MM) and other disorders, including immunoglobulin light-chain (AL) amyloidosis (5), anti-PLA2R antibody-positive membranous nephropathy (6), aplastic anemia with platelet transfusion refractoriness (7), antibody-mediated kidney transplant rejection (8, 9), and autoimmune diseases (10). Their indications continue to expand.

CD38, a type II transmembrane glycoprotein, is a receptor for CD31 in lymphocyte activation and proliferation (11, 12) and primarily an ectoenzyme for NAD^+^/NADP metabolism (13, 14), with production of Ca^2+^-mobilizing messengers (cADPR, ADPR, and NAADP) and regulation of intracellular NAD^+^ levels (15). It is also involved in adenosine production, which exerts immunosuppressive effects (16). Accumulating evidence indicates that CD38 promotes tumor cell proliferation and immune evasion. Its high expression in MM and other hematological tumors renders CD38 an important therapeutic target (17). It is also expressed at low levels on red blood cells (RBCs) (18). Therapeutic human IgG anti-CD38 monoclonal antibodies can bind to RBCs, leading to pan-agglutination in the indirect antiglobulin test and interfering with antibody screening and cross-matching (19). This interference may persist for months after therapy and poses transfusion safety challenges, particularly in MM patients who frequently require transfusion support (20, 21). The most commonly used solution to mitigate this interference is dithiothreitol (DTT) treatment, which disrupts the CD38 epitopes but also destroys several clinically significant blood group systems, including KEL, YT, DO, and VEL (3, 22, 23), and is ineffective for emerging targets like CD47, which is highly expressed on RBCs and is currently under investigation as a target in cancer therapy.

Alternative approaches to mitigate anti-CD38 interference have been reported, including modifications of anti-human globulin (AHG) detection using polyethylene glycol or polybromide (24). Strategies to block or absorb the anti-CD38 drug, such as soluble CD38 (20), anti-idiotype antibodies (20, 25), and Daudi cells (26, 27), as well as red blood cell-based approaches using anti-CD38 F(ab′)_2_ fragments (28, 29) or BMAP-treated RBCs (30), mask the CD38 antigen. However, these strategies have practical limitations that currently prevent their widespread use in routine clinical transfusion laboratories, as will be discussed in detail below.

To overcome the limitations of current methods, we developed an IgG-type rabbit anti-human CD38 mAb, D2. Its rabbit Fc region does not react with AHG, enabling it to mask CD38 on RBCs and eliminate DARA- or ISA-induced pan-agglutination in indirect antiglobulin tests (IATs) without affecting the detection of clinically significant irregular antibodies. Moreover, D2 is easy to use and compatible with routine workflows, providing a simple and effective solution to overcome anti-CD38 antibody interference.

## Materials and methods

### Ethical approval

The study was approved by the Ethics Committee of the Shaanxi Provincial Blood Center (No. 202518). Six-week-old female New Zealand White rabbits were purchased from Beijing Fuhao Experimental Animal Breeding Center. All animal experiments were approved by the Animal Welfare and Ethics Committee of Beijing Immune Ark Pharmaceutical Technology Co., Ltd. (No. IACUC2024-0022) and conducted in compliance with national animal welfare regulations.

### Cell lines

Human embryonic kidney 293 (HEK293) cells were procured from ATCC (Cat. CRL-1573) and cultured in OPM-293 CD05 serum-free medium (Shanghai OPM Biosciences Co., Ltd., Cat. 81075-001, Shanghai, China) at 36.5°C with 7.5% CO_2_ at 120 rpm in an oscillating incubator.

### Production of CD38 protein

The human CD38 gene vector (Sino Biological, NP_001766, Cat. HG10818-M, Beijing, China) was amplified using primers listed in **Supplementary Table S1**. PCR was used to amplify the extracellular region fragment of the human CD38 gene and a 6×His tag Val43-Ile300 in the reverse primer. The PCR product was gel-purified and cloned into the PQKX1 vector (Beijing Immunoah Pharma Tech Co., Ltd., Beijing, China), generating the PQKX1-hCD38-His construct. The plasmid was sequence-verified and transiently transfected into HEK293 cells using polyethyleneimine (PEI). After 7 days, supernatants were collected, and recombinant hCD38-His was purified with a HisTrap™ Excel column (GE Healthcare, Chicago, Illinois, USA), washed with 25 mM imidazole, eluted with 250 mM imidazole, and confirmed using sodium dodecyl sulfate-polyacrylamide gel electrophoresis (SDS-PAGE) with Coomassie brilliant blue staining.

### Production of rabbit anti-human CD38 polyclonal antibody

Six-week-old female New Zealand White rabbits were subcutaneously immunized with 0.5 mg hCD38-His over four doses. After the fourth immunization, cardiac blood was collected, and serum was separated via centrifugation and filtration. Antibodies were purified using a Protein A column (GE Healthcare, Chicago, Illinois, USA) and eluted with citrate buffer (pH 3.0). Purified antibodies were analyzed via Coomassie brilliant blue staining, and concentrations were determined using a NanoDrop spectrophotometer (Thermo Fisher Scientific, Waltham, MA, USA).

### Production of recombinant rabbit anti-human CD38 mAb

After four subcutaneous immunizations, New Zealand White rabbits were euthanized using CO_2_. CO_2_ was introduced into the euthanasia chamber at a flow rate of approximately 20%–30% of the chamber volume per minute, gradually increasing the concentration to ≥80% within 5 minutes. This concentration was maintained for an additional 5–10 minutes to ensure loss of consciousness and death. Following the confirmation of death, spleens were collected for single-cell suspension preparation and flow cytometric sorting. hCD38-His was labeled using an fluorescein isothiocyanate (FITC) Conjugation Kit (Fast)–Lightning-Link^®^ (Abcam, Cat. ab188285, Cambridge, Cambridgeshire, UK). For staining, 2 × 10^6^ splenocytes in 500 μL were incubated with 10 μg/mL FITC-hCD38-His and 5 μL Alexa Fluor^®^ 647 Donkey anti-rabbit IgG (BioLegend, Cat. 406414, San Diego, CA, USA) on ice for 15 minutes, washed, and stained with 5 μL of propidium iodide (PI; Sigma, Cat. 25535-16-4, St. Louis, Missouri, USA) for 5 minutes. Flow cytometric sorting was performed using Sony SH800 to isolate PI^−^/AF647^+^/FITC^+^ antigen-specific B cells into PCR tubes, frozen at −80°C for 2 minutes, centrifuged, and subjected to single-cell RT-PCR (Vazyme Biotech Co., Ltd., Cat. P621, Nanjing, Jiangsu Province, China) with IgG-specific heavy and light chain primers (Beijing Immunoah Pharma Tech Co., Ltd., Beijing, China). The PCR procedure is shown in **Supplementary Table S2**. Products (350 bp) were confirmed using agarose gel. The sequenced and verified products were used to synthesize the light and heavy chains, which were cloned into pQKR22 and pQKR23 vectors (Beijing Immunoah Pharma Tech Co., Ltd., Beijing, China), transfected into HEK293 cells, and purified using a Protein A column.

### Identification of recombinant hCD38-His, affinity evaluation of rabbit anti-CD38 polyclonal and monoclonal antibodies, and epitope competition with rabbit anti-CD38 mAbs

The affinity of rabbit anti-CD38 pAb and mAbs was evaluated using both ELISA and the ForteBio Octet system. For ELISA, hCD38-His was coated at 1 μg/mL. Rabbit pAb or mAbs were serially diluted in 15 steps, starting at 10 μg/mL for pAb and 20 μg/mL for mAbs, with phosphate-buffered saline (PBS) as blank. Bound antibodies were detected using horseradish peroxidase (HRP)-conjugated goat anti-rabbit IgG (Biodragon, Cat. BF03008, 1:5,000, Suzhou, Jiangsu Province, China), and absorbance at 450 nm was measured using a microplate reader. Binding affinity and dissociation kinetics between hCD38-His and rabbit anti-CD38 mAbs were assessed using the ForteBio Octet Red 96e system; hCD38-His was captured using Ni-NTA biosensors. Data were analyzed using GraphPad Prism.

ForteBio analysis was performed by first binding DARA (Janssen, Cat. PBS5P00, PA, USA) to CD38-His, followed by binding each of the two rabbit anti-CD38 monoclonal antibodies separately to determine whether these antibodies share overlapping epitopes with DARA.

### Flow cytometric analysis of rabbit anti-CD38 mAb binding to CD38 on human RBCs

Human RBCs (Shanghai Hemo Pharmaceutical & Biological Co., Ltd., Shanghai, China) were adjusted to a concentration of 5 × 10^7^ cells/mL. A rabbit mAb was diluted to 1,000 µg/mL and then serially diluted four times at a 1:9 ratio. For each dilution, 10 µL of antibody was added to 90 µL of RBC suspension. After thorough mixing, samples were incubated at 4°C for 1 hour, washed with PBS, and stained with FITC-conjugated goat anti-rabbit IgG at room temperature. Cells were resuspended in PBS and analyzed via flow cytometry.

### Evaluation of rabbit anti-human CD38 pAb and mAb in *in vitro* immunohematology testing

This study included 49 DARA- or ISA-treated patients. Residual ethylenediaminetetraacetic acid (EDTA) plasma and serum samples from 49 patients treated with anti-CD38 mAbs between 2024 and 2025 were stored at −80°C until analysis. To simulate anti-CD38 interference, antibody-negative plasma samples were spiked with 0.5 mg/mL DARA or ISA ISA (Sanofi, Cat. 4F026A, Paris, France), mimicking clinical conditions.

*In vitro* immunohematology testing of pAb and mAb was performed using the IAT. Unless otherwise stated, IATs were performed according to the manufacturer’s instructions using the Dexiang system. Briefly, 50 μL of 0.8% Reagent RBCs Surgiscreen^®^ (Ortho Clinical Diagnostics, Raritan, New Jersey, USA) and 40 μL of patient samples or commercial serum (with or without anti-CD38 antibodies) were added to an anti-human globulin detection card (Tianjin Dexiang Biotechnology Co., Ltd., Tianjin, China). Cards were incubated at 37°C for 15 minutes in K37-24 (Hangzhou Allsheng Instruments Co., Ltd., Hangzhou, Zhejiang Province, China) and centrifuged in an ID-centrifuge TD2-12 (Tianjin Dexiang Biotechnology Co., Ltd., Tianjin, China) using a two-phase program (55 g for 2 minutes and 200 g for 3 minutes).

Alternatively, IATs were performed in ID-Cards LISS/Coombs (Bio-Rad, Hercules, California, USA) according to the manufacturer’s recommended materials and procedures. Agglutination was visually assessed and graded from 0 to 4+, following the AABB Technical Manual: Interpretation of Agglutination Reactions, with scores assigned as 4+ (one solid agglutinate) = 12, 3+ (several large agglutinates) = 10, 2+ (medium agglutinates, clear background) = 8, 1+ (small agglutinates, turbid background) = 5, 1+w (very small agglutinates, turbid background) = 4, w+ or +/− (barely visible agglutination, turbid background) = 2, and 0 (no agglutination) = 0.

### Antigen masking IAT with rabbit anti-human CD38 pAb and mAbs

Rabbit pAb or mAb was diluted to a final concentration of 1 mg/mL. After centrifugation to remove the supernatant, 50 μL of rabbit pAb or mAb was added to 1 μL of packed RBCs and incubated at 37°C for 30 minutes. The cells were then resuspended in PBS to a final concentration of 0.8%. Subsequently, 50 μL of treated RBCs and 40 μL of serum containing DARA or ISA were added to an anti-human globulin card for an IAT, and agglutination was visually assessed.

### Optimal concentration and incubation time of recombinant rabbit anti-human CD38 mAb for eliminating DARA interference

Plasma samples negative for antibody screening were supplemented with 0.5 mg/mL DARA to evaluate the minimum concentration of rabbit mAb D2 needed to eliminate interference. Rabbit mAb was diluted to a final concentration of 1 mg/mL. After centrifugation, varying volumes of D2 (1–30 μL) were added to 1 μL of packed RBCs and incubated at 37°C for 30 minutes. The cells were then resuspended in PBS to a final concentration of 0.8%, followed by the IAT.

To optimize incubation time, 3 μL of 1 mg/mL D2 per μL of packed RBCs was incubated at room temperature for 15, 10, or 5 minutes. After resuspension to 0.8%, the treated RBCs were tested with 0.5 mg/mL DARA using the IAT.

### Comparison of the effects of D2 and DTT treatments on RBC blood group antigens

To compare the effects of DTT and optimized D2 treatment on major RBC antigen integrity, RBCs were processed using each method and tested for reactivity with a panel of blood group antibodies. DTT (Roche, Cat. 78655720, Basel, Switzerland) treatment was performed according to the protocol of the Austrian Society for Blood Group Serology, Transfusion Medicine, Regenerative Medicine, and Immunogenetics (ÖGBT), which is comparable to the DTT protocol described in Judd’s Methods in Immunohematology, 4th edition (31, 32). RBCs were washed four times with PBS, pH 7.4, and concentrated to 3%–5% in PBS, pH 7.4. Four volumes of 200 mM DTT (in PBS, pH 7.4) were added to one volume of cells. The mixture was incubated for 30 minutes at 37 °C, being dispersed gently from time to time. Afterward, the cells were washed four times with PBS, pH 7.4, and adjusted to 0.8% in PBS, pH 7.4. Non-hemolytic DTT-treated cells were used in standard IAT, and hemolytic cells were discarded (29). IgG reagents for anti-K, anti-k, anti-D, anti-S, anti-s, anti-Jk^a^, anti-Jk^b^, anti-Fy^a^, anti-Fy^b^, anti-Di^a^, and anti-Wr^a^ (CE Immundiagnostika GmbH, Germany) and anti-E, anti-e, anti-C, and anti-c (human IgG antibodies from our laboratory) were used. IgM reagents for anti-Le^a^ and anti-Le^b^ (Rapid Labs, Colchester, Essex, UK), and anti-M, anti-N, and anti-P1 (Shanghai Hemo Pharmaceutical & Biological Co., Ltd., Shanghai, China) were also applied. RBCs treated with either DTT or D2 were incubated with the antibodies. IgG antibodies were tested via the IAT using anti-human globulin detection cards; IgM antibodies were tested using the tube method at the immediate spin (IS) phase to compare antigen preservation.

### Detecting irregular antibodies in the presence of DARA and ISA

Plasma or serum samples were utilized, containing clinically relevant irregular antibodies such as IgG anti-D, anti-C, anti-E, anti-Fy^b^, anti-Jk^a^, anti-Jk^b^, anti-Le^a^, anti-Di^a^, anti-Di^b^, and anti-S, along with commercial serum samples containing IgG anti-K and anti-Fy^a^. Subsequently, DARA or ISA at a concentration of 0.5 mg/mL was introduced, followed by an IAT. The impact of optimized D2 on the detection of irregular antibodies was then assessed.

### Mitigating the DARA interference in clinical samples

Plasma samples from patients receiving DARA treatment were collected as described above and analyzed using the Reagent RBCs Surgiscreen^®^ and Makropanel 16 (Sanquin Reagents B.V., Amsterdam, Noord-Holland, Netherlands) to compare the effectiveness of DTT and optimized D2.

### Storage stability assessment of D2

To evaluate D2 storage stability, the antibody was sterile-filtered, aliquoted, and stored at 4°C, −20°C, or −80°C. After 1, 3, and 6 months, its ability to eliminate DARA-induced interference in the IAT was tested.

## Results

### Production and identification of recombinant hCD38-His and rabbit anti-human CD38 pAb

**Figure 1:**
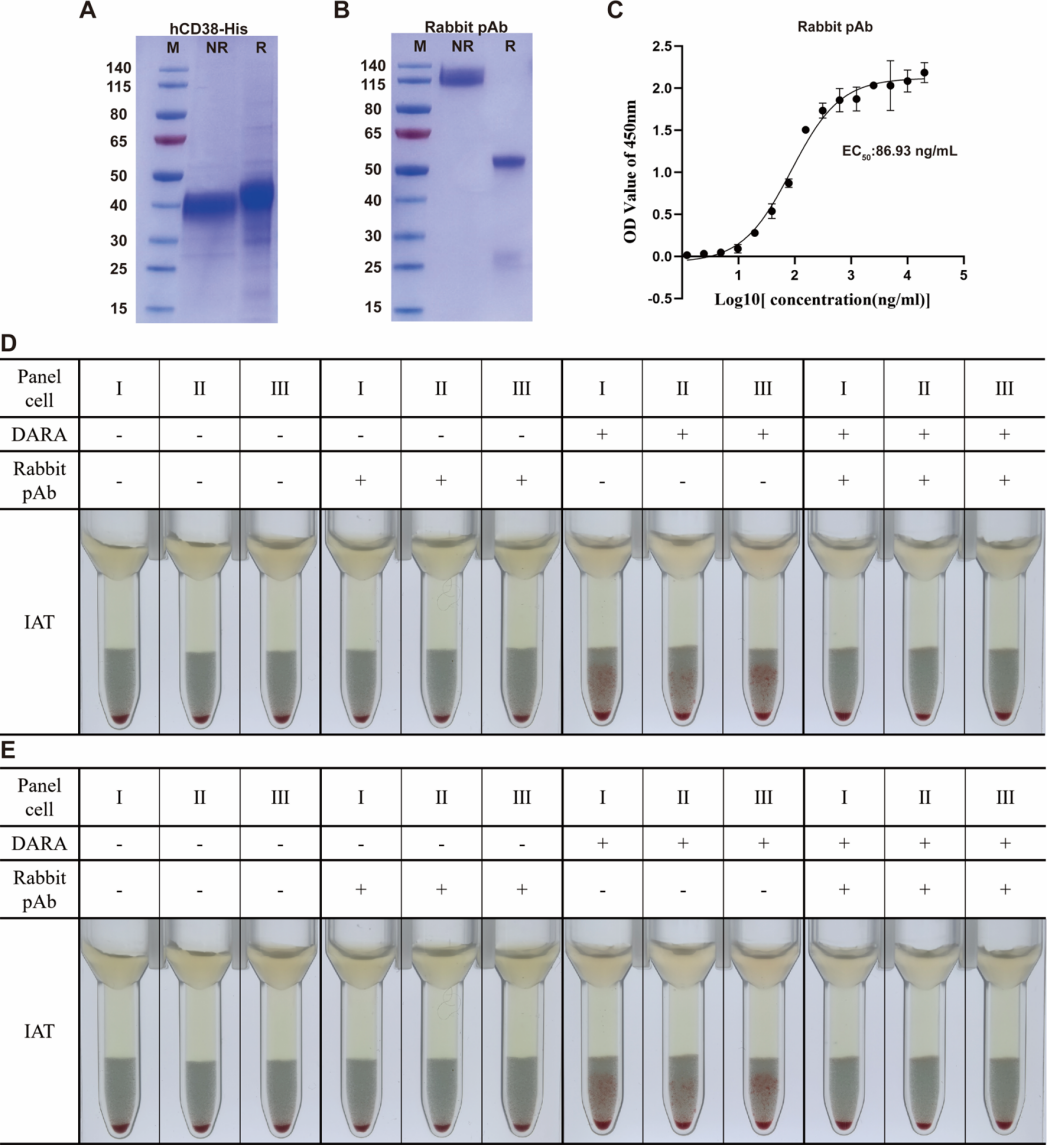
Expression, characterization, and functional evaluation of rabbit anti-hCD38 polyclonal antibody. **(A)** Purity and molecular weight of hCD38-His protein detected via SDS–PAGE and Coomassie brilliant blue staining. **(B)** Purity and molecular weight of rabbit anti-hCD38 pAb analyzed via SDS–PAGE and Coomassie staining. **(C)** Affinity of rabbit pAb to hCD38-His determined via ELISA (EC_50_ = 86.93 ng/mL). **(D)** Results of indirect anti-human globulin tests in the presence (plus sign) or absence (minus sign) of DARA and pAb are shown. pAb-treated RBCs eliminated DARA-induced pan-agglutination in IAT. **(E)** Same as panel **(D)**, using ISA-spiked plasma. The results showed that treatment with pAb eliminated ISA-induced pan-agglutination in IAT. Rabbit pAb, rabbit anti-human CD38 polyclonal antibody; DARA, daratumumab; ISA, isatuximab; pAb, polyclonal antibody; RBCs, red blood cells; IAT, indirect antiglobulin test. A solid pellet at the bottom of the tubes indicates a negative result, and suspended particles (red cell agglutinates) within the gel matrix indicate a positive test result (either a 1+ or 2+ degree of agglutination).

The human CD38 gene was amplified via PCR and cloned into vectors to generate CD38-His protein. SDS–PAGE confirmed the expected molecular weight and purity of hCD38-His (**Figure 1A**). Rabbits were immunized with CD38-His, and sera were collected and purified to obtain rabbit pAb. SDS–PAGE confirmed the expected molecular weight and purity of rabbit pAb (**Figure 1B**). Affinity was evaluated via ELISA. The EC_50_ was 86.93 ng/mL, confirming that the rabbit pAb binds hCD38-His with high affinity (**Figure 1C**).

To evaluate functional efficacy, we assessed whether the rabbit pAb could block interference from therapeutic anti-CD38 mAbs, thereby restoring IAT specificity. We tested its ability to block interference from two commonly used CD38 mAbs, DARA and ISA, at a concentration of 0.5 mg/mL. We evaluated the rabbit polyclonal antibody’s interference-eliminating ability against therapeutic CD38 at 0.5 mg/mL using IATs. As shown in **Figures 1D, E**, RBCs treated with rabbit pAb effectively eliminated the pan-agglutination caused by interference from DARA and ISA.

### The production and identification of rabbit anti-human CD38 mAb

**Figure 2:**
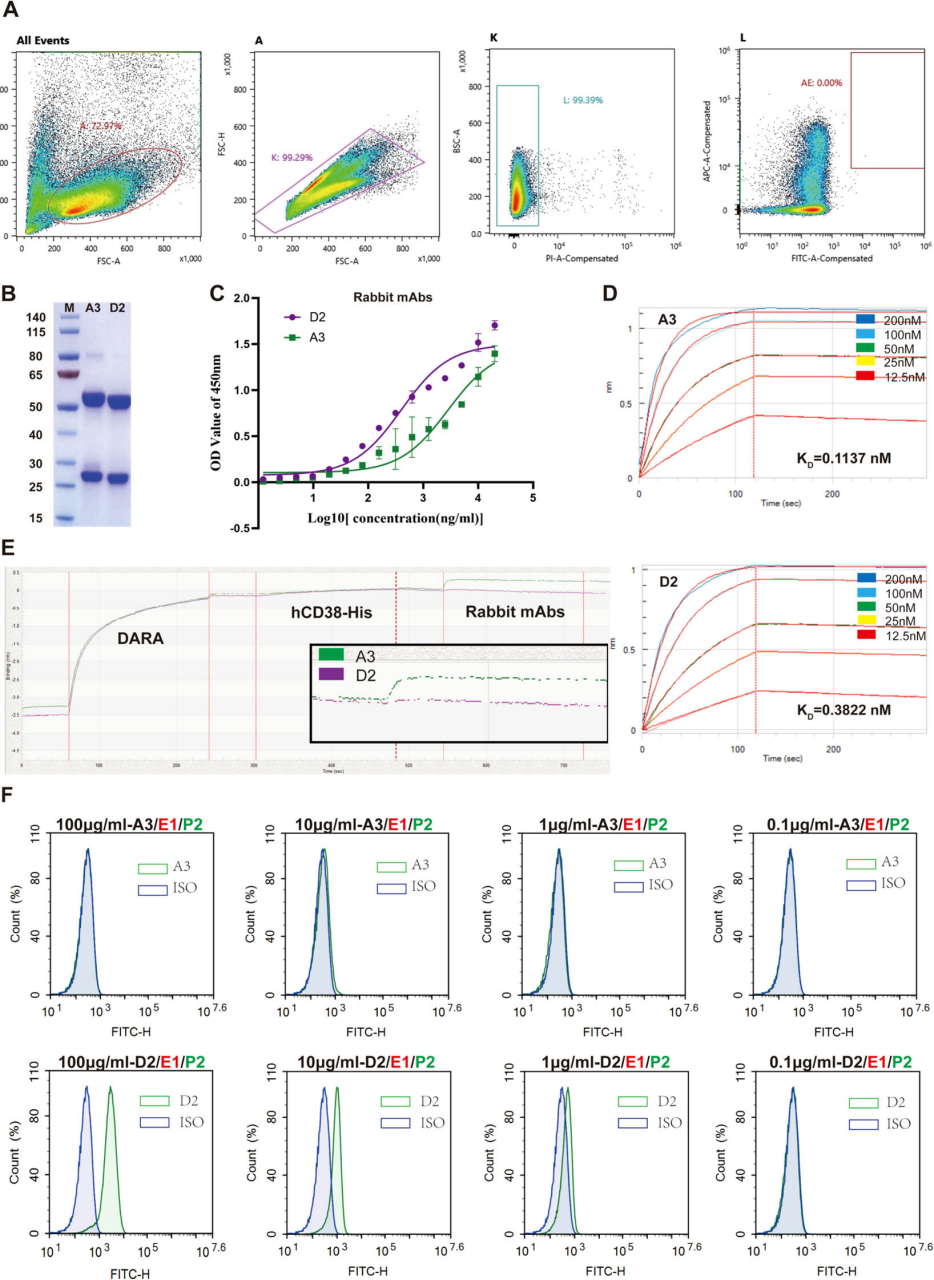
Preparation, expression, and identification of rabbit anti-human CD38 monoclonal antibodies. **(A)** Flow cytometric sorting of single B cells; FITC/allophycocyanin (APC) (AF647) double-positive cells in AE gate selected. **(B)** Purity and molecular weight of rabbit mAbs were detected via staining with Coomassie brilliant blue. **(C)** ELISA analysis of rabbit mAb binding to hCD38-His. **(D)** ForteBio Octet determination of the affinities of rabbit mAbs toward hCD38-His. The binding affinity parameter KD was calculated, as reported using ForteBio Data Analysis Software 8.0 (Fremont, CA, USA). **(E)** Epitope competition assay with DARA by ForteBio. Green, A3; purple, D2. **(F)** Flow cytometry showing D2 and A3 binding to RBC-expressed CD38. Rabbit mAbs, rabbit anti-human CD38 monoclonal antibodies; DARA, daratumumab; RBC, red blood cell.

After confirming that rabbit anti-human CD38 pAb could eliminate interference induced by therapeutic CD38 mAbs, we planned to use rabbit mAbs with similar activity. Spleen cells were harvested from euthanized rabbits, and single antigen-specific B cells (PI^−^/AF647^+^/FITC^+^) were isolated via fluorescence-activated cell sorting (FACS) (**Figure 2A**). RT-PCR, cloning, sequencing, and expression in HEK293 cells yielded two rabbit mAbs, D2 and A3. SDS–PAGE confirmed their expected molecular weights and purity (**Figure 2B**).

We next assessed the functional characteristics of D2 and A3. ELISA confirmed the dose-dependent binding of both mAbs to hCD38-His across a concentration range of 20 to 20,000 ng/mL (**Figure 2C**). We used ForteBio Octet to confirm the binding of each rabbit’s mAbs to hCD38-His, with dissociation constants (KD) of 0.3822 nM for D2 and 0.1137 nM for A3 (**Figure 2D**). To evaluate potential epitope overlap between rabbit mAbs and therapeutic anti-CD38 mAb, we performed a competitive binding assay using the ForteBio system. If epitope overlap existed, the binding of the rabbit antibodies would be competitively inhibited. As shown in **Figure 2E**, D2 was unable to bind to hCD38-His after DARA saturation, indicating that D2 and DARA likely recognize overlapping epitopes. In contrast, A3 retained its ability to bind hCD38-His following DARA pre-binding, suggesting that A3 targets a distinct, non-overlapping epitope on the CD38 protein. To further evaluate the ability of D2 and A3 to recognize native CD38 expressed on human RBCs, we performed flow cytometric analysis. The results demonstrated that D2 bound to native CD38 on RBCs in a dose-dependent manner, whereas A3 showed minimal or no binding (**Figure 2F**).

**Figure 3:**
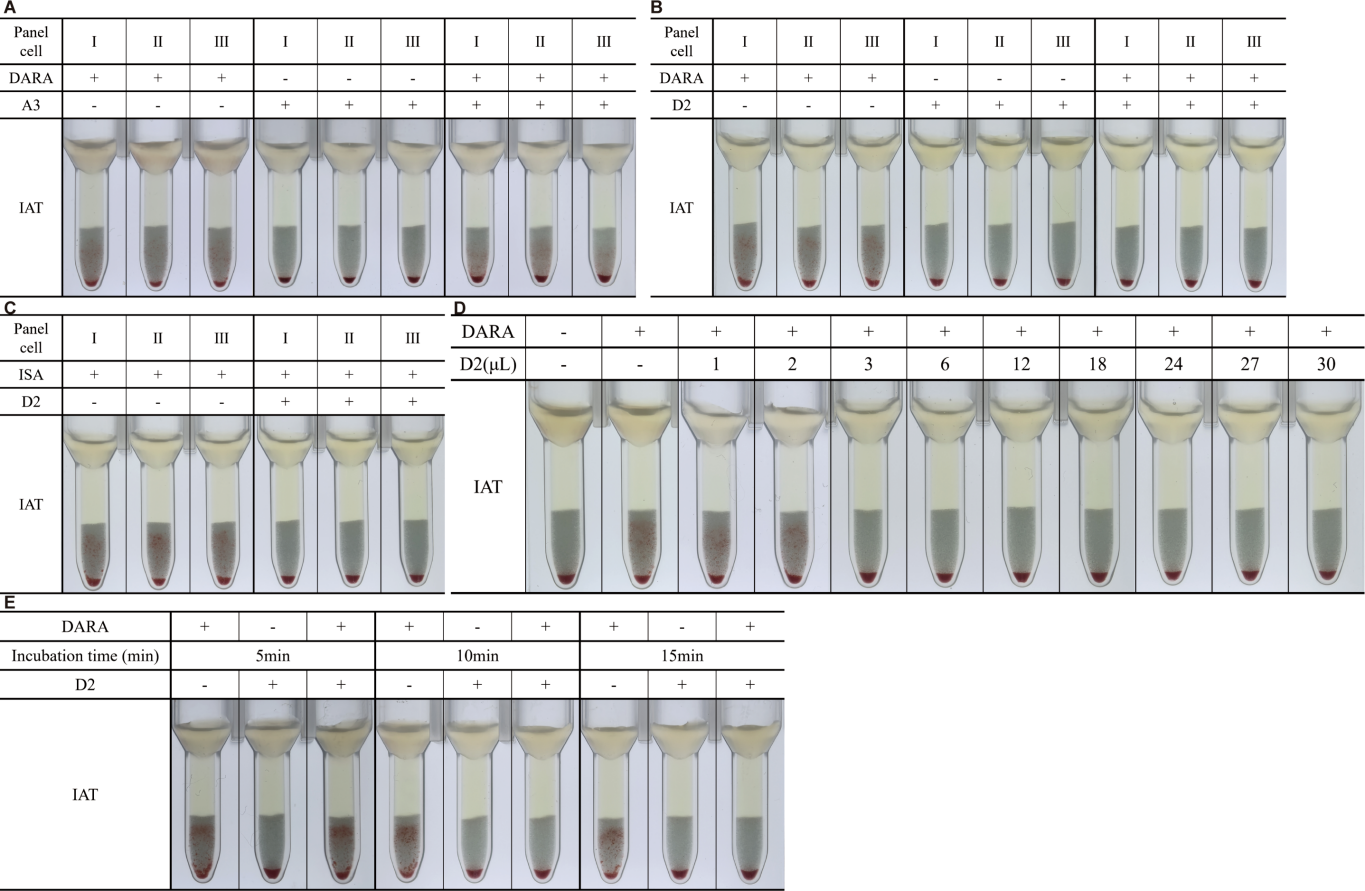
Elimination of DARA- and ISA-induced interference in IAT using rabbit anti-human CD38 monoclonal antibodies and optimization of D2 treatment conditions. Results of indirect anti-human globulin tests in the presence (plus sign) or absence (minus sign) of DARA/ISA and A3/D2 are shown. **(A)** A3-treated RBCs failed to eliminate DARA-induced interference in IAT. **(B, C)** D2-treated RBCs eliminated DARA- and ISA-induced pan-agglutination in IAT. **(D)** Tube 1: negative control. Tube 2: positive control for the DARA interference. Tubes 3–9: per μL of packed RBCs was treated with varying volumes of 1–30 μL D2 (1 mg/mL), and at least 3 μL of 1 mg/mL D2 was required to effectively eliminate DARA interference. **(E)** Per μL packed RBCs was treated with 3 μL of D2 at room temperature for varying durations (5–15 minutes). Optimization experiments demonstrated that an incubation time of 10 minutes at room temperature was sufficient to eliminate DARA interference in the presence of D2. DARA, daratumumab; ISA, isatuximab; IAT, indirect antiglobulin test; RBCs, red blood cells. A solid pellet at the bottom of the tubes indicates a negative result, and suspended particles (red cell agglutinates) within the gel matrix indicate a positive test result (either a 1+ or 2+ degree of agglutination).

To further assess the ability of D2 and A3 to eliminate interference from therapeutic anti-CD38 mAbs, an IAT was performed. As shown in **Figure 3A**, the A3 treatment failed to eliminate the agglutination. D2 treatment effectively eliminated pan-agglutination induced by both DARA and ISA (**Figures 3B, C**), indicating that only D2 exhibited functional blocking capacity against anti-CD38 mAb-induced interference.

### Optimal concentration and incubation time of D2 for eliminating DARA interference

To determine the minimum effective concentration of D2 required to eliminate DARA interference, 1 μL of packed RBCs was incubated with 1–30 μL D2 (1 mg/mL), followed by an IAT. As shown in **Figure 3D**, at least 3 μL of 1 mg/mL D2 antibody was necessary to eliminate DARA-induced agglutination.

To optimize incubation time, 1 μL of packed RBCs was treated with 3 μL of D2 at room temperature for varying durations (5–15 minutes), followed by the addition of DARA testing and IAT. As shown in **Figure 3E**, a 10-minute incubation was sufficient to eliminate DARA-induced interference. Therefore, all subsequent experiments were performed using 1 μL of packed RBCs treated with 3 μL of 1 mg/mL D2 for 10 minutes.

### Effect of recombinant rabbit anti-human CD38 mAb on RBC blood group antigens

**Figure 4:**
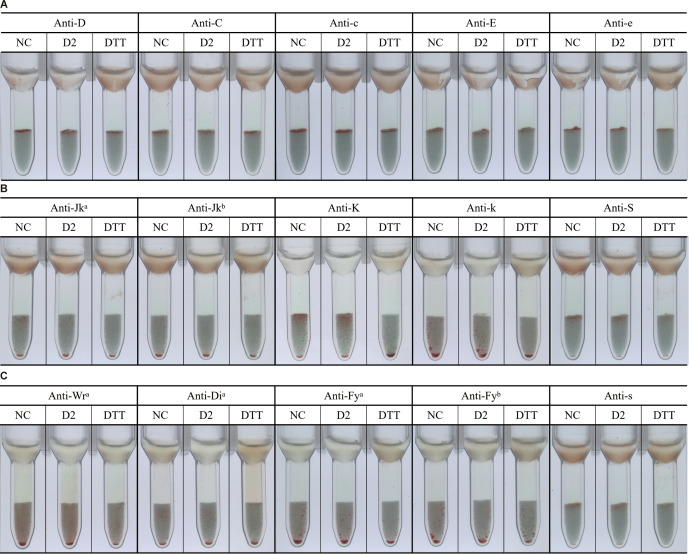
Elimination of DARA- and ISA-induced interference in IAT using rabbit anti-human CD38 monoclonal antibodies and optimization of D2 treatment conditions. Results of indirect anti-human globulin tests in the presence (plus sign) or absence (minus sign) of DARA/ISA and A3/D2 are shown. **(A)** A3-treated RBCs failed to eliminate DARA-induced interference in IAT. **(B, C)** D2-treated RBCs eliminated DARA- and ISA-induced pan-agglutination in IAT. **(D)** Tube 1: negative control. Tube 2: positive control for the DARA interference. Tubes 3–9: per μL of packed RBCs was treated with varying volumes of 1–30 μL D2 (1 mg/mL), and at least 3 μL of 1 mg/mL D2 was required to effectively eliminate DARA interference. **(E)** Per μL packed RBCs was treated with 3 μL of D2 at room temperature for varying durations (5–15 minutes). Optimization experiments demonstrated that an incubation time of 10 minutes at room temperature was sufficient to eliminate DARA interference in the presence of D2. DARA, daratumumab; ISA, isatuximab; IAT, indirect antiglobulin test; RBCs, red blood cells. A solid pellet at the bottom of the tubes indicates a negative result, and suspended particles (red cell agglutinates) within the gel matrix indicate a positive test result (either a 1+ or 2+ degree of agglutination).

A major limitation of DTT treatment is that it destroys CD38 as well as several clinically significant blood group antigens, most notably KEL1 (K) and KEL2 (k) (3, 22, 23). To evaluate whether D2 affects RBC antigen integrity, we compared the antigen reactivity of untreated RBCs, optimized D2-treated RBCs, and DTT-treated RBCs against a panel of blood group antibodies targeting antigens including D, C, c, E, e, M, N, S, s, P1, Jk^a^, Jk^b^, K, k, Wr^a^, Di^a^, Fy^a^, Fy^b^, Le^a^, and Le^b^. The IAT with antiglobulin gel cards (**Figures 4A–C**) and the tube method at the IS phase (**Supplementary Figures S1A, B**) showed that D2-treated RBCs did not affect the expression of those tested blood group antigens, comparable to untreated RBCs. In contrast, DTT-treated RBCs showed loss of K and k expression. These results indicate that D2 treatment preserves the antigenic integrity of RBCs, offering a clear advantage over DTT in pretransfusion testing.

### Rabbit anti-human CD38 mAb effectively eliminates interference of anti-CD38 therapeutic antibodies without affecting the detection of irregular antibodies

**Figure 5:**
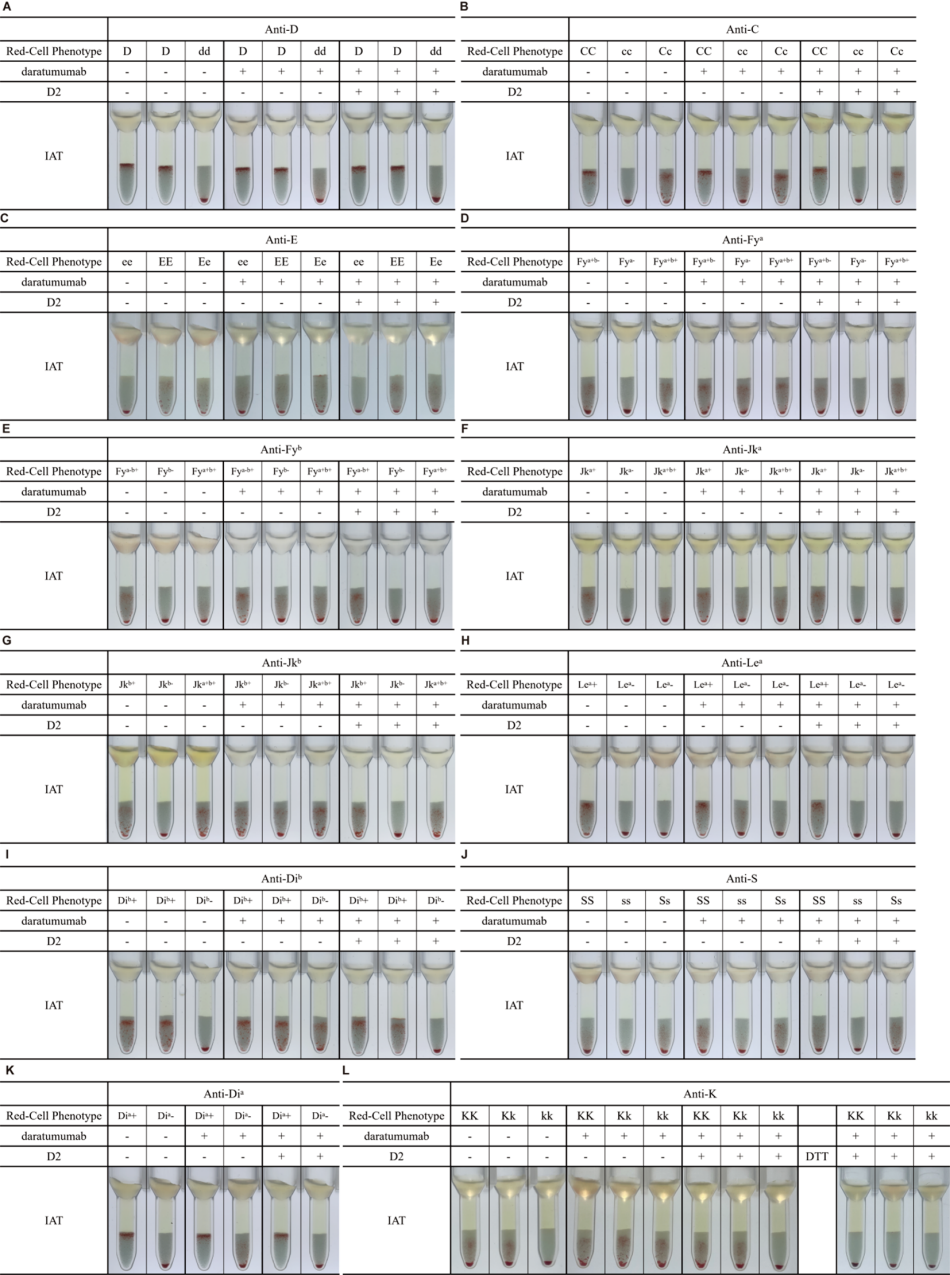
D2-treated eliminates the interference caused by DARA without affecting the detection of irregular antibodies. Results of indirect anti-human globulin (Coombs’) tests of red cell antibody-positive plasma specimens in the presence (plus sign) or absence (minus sign) of daratumumab and D2 are shown. Plasma or serum specimens obtained from patients with known red cell irregular antibodies, or from commercial serum samples, were spiked with daratumumab to a final concentration of 0.5 mg/mL and then tested. **(A)** IAT results showing that D2-treated RBCs enabled detection of anti-D in plasma containing DARA or **(B)** anti-C, **(C)** anti-E, **(D)** anti-Fy^a^, **(E)** anti-Fy^b^, **(F)** anti-Jk^a^, **(G)** anti-Jk^b^, **(H)** anti-Le^a^, **(I)** anti-Di^b^, **(J)** anti-S, or **(K)** anti-Di^a^. **(L)** Comparison of IAT results for anti-K detection using D2-treated versus DTT-treated RBCs in plasma containing DARA. A solid pellet at the bottom of the tubes indicates a negative result, and suspended particles (red cell agglutinates) within the gel matrix indicate a positive test result (either a 1+ or 2+ degree of agglutination). DARA, daratumumab; IAT, indirect antiglobulin test; RBCs, red blood cells; DTT, dithiothreitol.

We obtained plasma or serum samples of known clinically relevant irregular antibodies (IgG anti-D, anti-C, anti-E, anti-Fy^b^, anti-Jk^a^, anti-Jk^b^, anti-Le^a^, anti-Di^a^, anti-Di^b^, and anti-S) and commercial antisera for IgG anti-K and anti-Fy^a^. DARA or ISA was added to each sample at a concentration of 0.5 mg/mL. To evaluate whether D2-treated RBCs could effectively eliminate DARA or ISA interference in the IAT while preserving the detection of irregular antibodies, D2-treated RBCs were incubated with these antibody-containing samples. As shown in **Figures 5A–L**, D2-treated RBCs eliminated pan-agglutination caused by DARA in the IAT while maintaining reactivity with all tested irregular antibodies. Comparable results were also observed in samples containing ISA, as shown in **Supplementary Figures S2A–L**. These findings confirm that D2 treatment eliminates therapeutic anti-CD38 mAb interference without compromising irregular antibody detection in pretransfusion testing.

### Mitigating the anti-CD38 mAb interference in clinical samples

**Figure 6:**
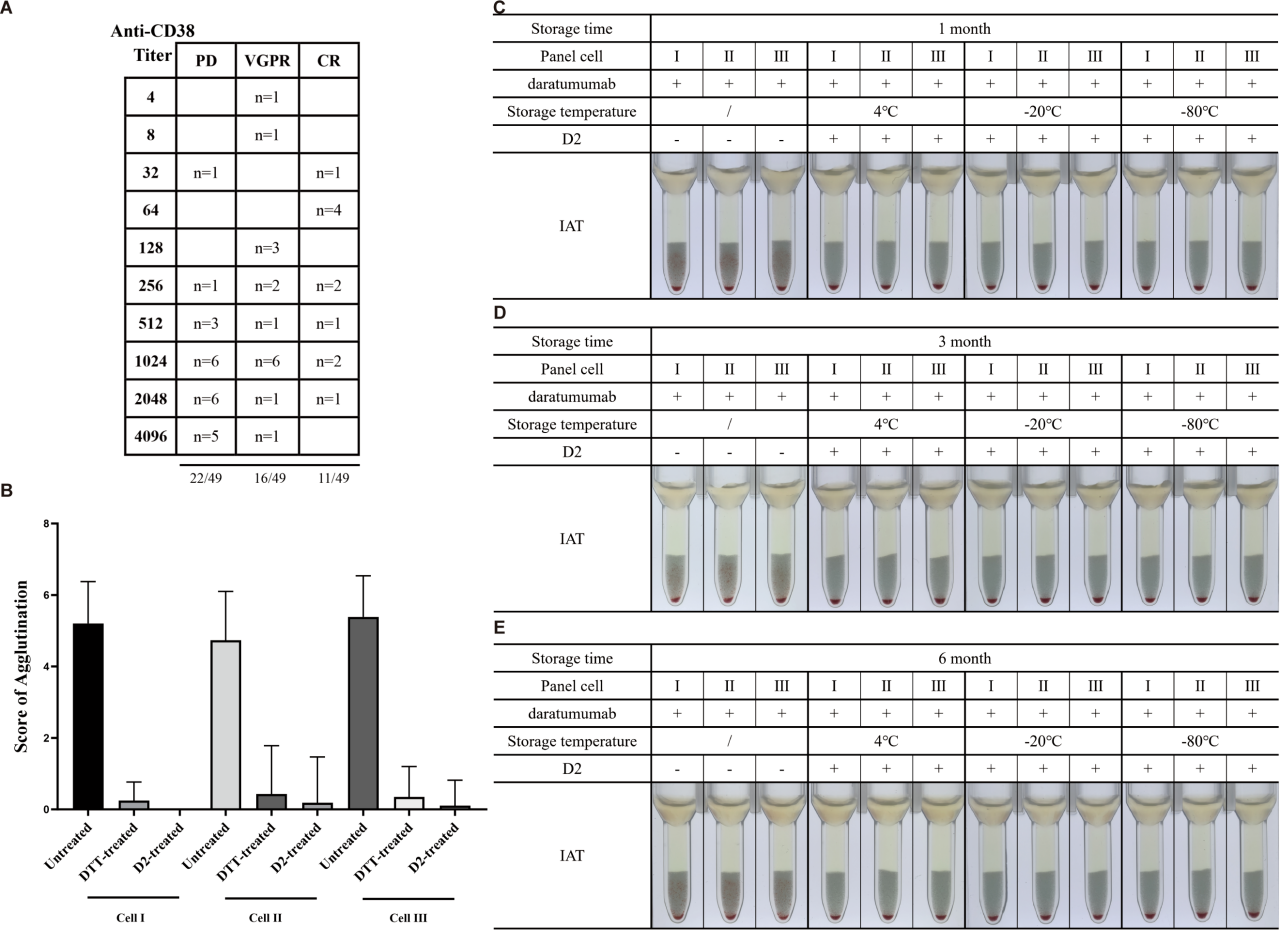
Stability of D2 and its efficacy in eliminating therapeutic anti-CD38 antibody interference. Results of indirect anti-human globulin (Coombs’) tests in the presence (plus sign) or absence (minus sign) of daratumumab and D2 are shown. **(A)** Distribution of anti-CD38 antibody titers (n = 49) and their association with clinical response status. **(B)** Agglutination scores comparing the efficacy of DTT and D2 in reducing therapeutic anti-CD38 antibody interference. **(C)** To evaluate the storage stability of D2, it was stored at 4°C, −20°C, and −80°C, and its ability to eliminate DARA interference was assessed via IAT after 1 month **(D)**, 3 months **(E)**, or 6 months. A solid pellet at the bottom of the tubes indicates a negative result, and suspended particles (red cell agglutinates) within the gel matrix indicate a positive test result (either a 1+ or 2+ degree of agglutination). DTT, dithiothreitol; DARA, daratumumab; IAT, indirect antiglobulin test.

A total of 49 patients receiving DARA therapy were included, of whom 45 (91.8%) had multiple myeloma, two had T-lymphoblastic leukemia/lymphoma, one had non-Hodgkin lymphoma, and one had myelodysplastic syndromes. Since ISA was only approved by the Chinese NMPA on January 8, 2025, only two clinical samples from ISA-treated patients were available. Antibody titration of all 49 patient plasma samples showed titers ranging from 4 to 4,096, with over half (33 patients, 67%) exhibiting titers ≥512. Among these 33 patients, 20 were experiencing progressive disease (PD) following anti-CD38 therapy, and nine of these 20 samples were not fully cleared of interference after DTT treatment (**Figure 6A**).

To assess D2 efficacy, we compared IAT results of untreated, optimized D2-treated, and DTT-treated RBCs with patient samples. As shown in **Table 1**, **Figure 6B**, D2 effectively eliminated DARA interference in all samples. In contrast, DTT treatment resulted in 79% fully negative reactions, with 10 samples still showing some agglutination. Some agglutination remained in D2-treated RBCs for Cell and Cell III; this residual reaction corresponds to the presence of specific antibodies. For patients 9 and 24, interference removal validation revealed positive antibody screening. Further testing using D2-treated RBCs for antibody identification demonstrated the presence of IgG anti-Wr^a^ in patient 9 and IgG anti-E in patient 24. These results indicate that D2 treatment is more effective than DTT in eliminating anti-CD38 mAb interference while preserving the detection of clinically relevant antibodies.

**Table 1:** D2 mitigates interference of anti-CD38 mAbs in clinical samples.

Patient	Age (years), gender	Diagnosis	Anti-CD38 mAb	Days since last known anti-CD38 dose (n)	Response to anti-CD38 mAb (sCR, CR, VGPR, or PD)	Anti-CD38 titer	Treatment	Agglutination (AHG: 0–4+)		
								Cell I	Cell II	Cell III
1	67, M	MM	DARA	2	PD	1,024	None	1+	1+	1+
							D2	0	0	0
							DTT	± w	± w	± w
2	66, M	MM	DARA	7	VGPR	512	None	1+	1+	1+
							D2	0	0	0
							DTT	0	0	0
3	54, F	MM	DARA	13	CR	64	None	1+	1+	1+
							D2	0	0	0
							DTT	0	0	0
4	61, M	MM	DARA	1	PD	4,096	None	1+	1+	1+s
							D2	0	0	0
							DTT	0	0	0
5	61, M	MM	DARA	15	CR	256	None	1+	1+	1+
							D2	0	0	0
							DTT	0	0	0
6	78, M	MM	DARA	32	VGPR	4	None	±	± w	±
							D2	0	0	0
							DTT	0	0	0
7	66, M	MM	DARA	28	CR	32	None	1+	1+w	1+
							D2	0	0	0
							DTT	0	0	0
8	67, F	MM	DARA	13	CR	64	None	1+	1+	1+
							D2	0	0	0
							DTT	0	0	0
9^a^	72, M	MM	DARA	79	VGPR	8	None	±	±	1+
							D2	0	0	1+
							DTT	0	0	1+
10	65, F	MM	DARA	14	CR	64	None	1+	1+	1+
							D2	0	0	0
							DTT	0	0	0
11	81, M	MM	DARA	13	VGPR	1,024	None	1+	1+	1+
							D2	0	0	0
							DTT	0	0	0
12	61, M	MM	DARA	32	VGPR	128	None	1+	1+	1+
							D2	0	0	0
							DTT	0	0	0
13	61, M	MM	DARA	5	PD	4,096	None	2+	1+s	2+
							D2	0	0	0
							DTT	0	0	0
14	84, F	MM	DARA	34	CR	512	None	1+	1+	1+
							D2	0	0	0
							DTT	0	0	0
15	70, M	MM	DARA	18	VGPR	128	None	1+	1+	1+
							D2	0	0	0
							DTT	0	0	0
16	35, M	MM	DARA	8	CR	1,024	None	1+	1+	1+
							D2	0	0	0
							DTT	0	0	0
17	74, M	MM	DARA	14	CR	64	None	1+	1+	1+
							D2	0	0	0
							DTT	0	0	0
18	74, F	MM	DARA	11	PD	2,048	None	1+	1+	1+
							D2	0	0	0
							DTT	0	0	0
19	65, F	MM	DARA	14	PD	2,048	None	1+s	1+	1+s
							D2	0	0	0
							DTT	0	0	0
20	63, F	MM	DARA	28	PD	256	None	1+	1+	1+
							D2	0	0	0
							DTT	± w	± w	± w
21	68, M	MM	DARA	34	CR	256	None	1+	1+	1+
							D2	0	0	0
							DTT	0	0	0
22	65, F	MM	DARA	2	PD	512	None	1+	1+	1+
							D2	0	0	0
							DTT	±	±	±
23	31, F	NHL	DARA	9	PD	1,024	None	1+	1+	1+
							D2	0	0	0
							DTT	0	0	0
24^b^	73, F	MM	DARA	18	PD	512	None	1+	2+s	1+
							D2	0	2+s	0
							DTT	0	2+s	0
25	50, M	MM	DARA	2	PD	1,024	None	1+	1+	1+
							D2	0	0	0
							DTT	± w	± w	± w
26	75, F	MM	DARA	10	VGPR	1,024	None	1+	1+	1+
							D2	0	0	0
							DTT	0	0	0
27	81, F	MM	DARA	7	VGPR	1,024	None	1+	1+	1+
							D2	0	0	0
							DTT	0	0	0
28	79, F	MM	DARA	3	VGPR	1,024	None	1+	1+	1+
							D2	0	0	0
							DTT	0	0	0
29	54, M	MDS	DARA	7	VGPR	4,096	None	1+	1+	1+
							D2	0	0	0
							DTT	0	0	0
30	64, M	MDS	DARA	25	VGPR	128	None	1+	1+	1+
							D2	0	0	0
							DTT	0	0	0
31	61, F	MM	DARA	8	VGPR	256	None	1+	1+	1+
							D2	0	0	0
							DTT	0	0	0
32	56, M	MM	DARA	4	PD	1,024	None	1+	1+	1+
							D2	0	0	0
							DTT	0	0	0
33	74, F	MM	DARA	16	PD	1,024	None	1+	1+	1+
							D2	0	0	0
							DTT	0	0	0
34	62, M	MM	DARA	22	VGPR	256	None	1+	1+	1+
							D2	0	0	0
							DTT	0	0	0
35	59, M	MM	DARA	2	PD	2,048	None	1+	1+s	1+s
							D2	0	0	0
							DTT	0	0	0
36	69, M	MM	DARA	15	VGPR	1,024	None	1+	1+	1+
							D2	0	0	0
							DTT	0	0	0
37	65, M	MM	DARA	4	VGPR	2,048	None	1+	1+	1+
							D2	0	0	0
							DTT	0	0	0
38	73, M	MM	DARA	5	VGPR	1,024	None	1+	1+	1+s
							D2	0	0	0
							DTT	0	0	0
39	59, M	MM	DARA	40	CR	2,048	None	1+	±	1+
							D2	0	0	0
							DTT	0	0	0
40	63, F	MM	DARA	44	PD	2,048	None	1+s	1+	1+s
							D2	0	0	0
							DTT	± w	± w	± w
41	60, M	MM	DARA	91	CR	1,024	None	1+	±	1+
							D2	0	0	0
							DTT	0	0	0
42	65, M	MM	DARA	2	PD	2,048	None	1+s	1+	1+s
							D2	0	0	0
							DTT	± w	± w	± w
43	15, F	T-LBL/ALL	DARA	3	PD	2,048	None	1+s	1+	1+s
							D2	0	0	0
							DTT	0	0	0
44	34, M	T-LBL/ALL	DARA	4	PD	1,024	None	1+	±	1+
							D2	0	0	0
							DTT	± w	± w	± w
45	52, M	MM	ISA	4	PD	32	None	±	± w	±
							D2	0	0	0
							DTT	0	0	0
46	52, F	MM	DARA	12	PD	4,096	None	1+s	1+	1+s
							D2	0	0	0
							DTT	± w	± w	± w
47	82, M	MM	DARA	29	PD	4,096	None	1+s	1+	1+s
							D2	0	0	0
							DTT	± w	± w	± w
48	59, F	MM	ISA	15	PD	4,096	None	1+s	1+	1+s
							D2	0	0	0
							DTT	± w	± w	± w
49	83, M	MM	DARA	30	PD	512	None	1+s	1+	1+s
							D2	0	0	0
							DTT	0	0	0

anti-CD38 mAb, anti-CD38 monoclonal antibody; AHG, anti-human globulin; D2, rabbit anti-human CD38 antibody; DTT, 0.2 M dithiothreitol; DARA, daratumumab; ISA, isatuximab; MDS, myelodysplastic syndrome; MM, multiple myeloma; T-LBL/ALL, T-lymphoblastic leukemia/lymphoma; sCR, stringent complete response; CR, complete response; VGPR, very good partial response; PD, progressive disease.

a Identification of patient 9 confirmed the presence of anti-Wr^a^.

b Identification of patient 24 confirmed the presence of anti-E.

### D2 maintains mitigating activity against DARA after 6 months at 4°C

As shown in **Figures 6C–E**, the IAT demonstrated that D2-treated RBCs stored at 4°C for up to 6 months retained the ability to eliminate DARA-induced interference. These findings indicate that D2 remains stable and functionally effective under standard refrigeration conditions, highlighting its high clinical application value.

## Discussion

Globally, the number of patients receiving CD38-targeted therapies—particularly DARA and ISA—continues to rise, making CD38-related serologic interference an increasingly routine challenge for immunohematology laboratories. As the therapeutic indications for anti-CD38 mAbs broaden, the complexity of antibody screening and identification in transfusion testing is expected to increase accordingly. This trend underscores the urgent need for a mitigation strategy that is safe, efficient, and compatible with routine laboratory workflows.

Currently, the most widely used approach to eliminate CD38 mAb interference involves treating reagent RBCs with DTT (3, 20, 25, 28, 30, 33, 34), thus carrying the risk of missing clinically significant irregular antibodies. Moreover, it is technically demanding and time-consuming and affects the stability of RBCs, limiting its practical applicability in routine settings. Current alternative strategies to DTT, for instance, replacing the IAT with polyethylene glycol or polybromide assays, can circumvent CD38 mAb interference. Nevertheless, these methods may lack sensitivity for low-titer or low-affinity irregular antibodies, potentially resulting in false-negative outcomes (24). Other reported approaches, while theoretically promising, also face practical constraints. Soluble CD38 (sCD38) may interfere with anti-Fy antibodies and is costly and not readily available and would require large amounts to neutralize therapeutic monoclonal antibodies (19, 20); anti-idiotype antibodies against DARA are specific only to DARA and are ineffective against other CD38-targeting agents (35); the use of cells with high CD38 expression to neutralize or adsorb therapeutic anti-CD38 antibodies, although partially effective in mitigating interference, may dilute patient serum antibodies and compromise the detection of weak irregular antibodies. Moreover, these cells are difficult to preserve and require cumbersome handling procedures, which limit their practical applicability (26); BMAP enzymatic treatment is technically demanding and time-consuming (30). Anti-CD38 F(ab′)_2_ fragments offer a more targeted alternative by masking CD38 epitopes on RBCs, thereby preventing the binding of therapeutic antibodies (28). The commercial reagent DaraEx plus, which contains a Fab fragment of an anti-CD38 antibody, has demonstrated clear efficacy in reducing interference from daratumumab, isatuximab, felzartamab, and mezagitamab (29, 36). However, DaraEx plus is limited to certain gel card systems (29). DaraEx plus was not available to us during the study period and therefore could not be included for direct comparison in the present work.

In this study, we developed a novel rabbit anti-human CD38 antibody, D2, which can be stably produced, is simple to use, and effectively eliminates interference. D2 competitively binds to CD38 on RBCs, thereby blocking DARA- or ISA-induced interference without altering the expression of other clinically significant antigens. This AMIAT strategy preserves RBC blood group antigens (36), does not compromise the detection of irregular antibodies, and restores the integrity of the IAT. Its ease of use, antigen-preserving properties, and compatibility with routine blood bank workflows make D2 a promising tool for clinical implementation. In addition to D2, we also obtained another mAb, A3. Although A3 was able to bind recombinant hCD38-His, it did not compete with DARA for binding to the same epitope on hCD38-His, and importantly, it failed to bind CD38 expressed on the surface of human RBCs. The inability of A3 to recognize native RBC-associated CD38 may be due to conformational differences between recombinant CD38 and the native membrane-bound form, as well as the possibility that the A3 epitope is located close to the RBC membrane, where steric hindrance prevents effective binding in a cellular context. Consistent with this, serologic testing confirmed that A3 was unable to neutralize DARA-induced interference in the IAT, further supporting its lack of interaction with native CD38 on RBCs.

The use of single B-cell screening and recombinant production to generate a stable D2 cell line ensures scalable manufacturing for clinical pretransfusion testing. At a low working concentration (3 μL of 1 mg/mL per 1 μL of packed RBCs), D2 rapidly (within 10 minutes) blocks pan-agglutination caused by both antibodies without requiring wash steps, thereby improving workflow efficiency. Its strong compatibility with existing workflows enables seamless integration into routine antibody screening and cross-matching procedures in hospital transfusion services and blood group laboratories, facilitating clinical implementation.

In clinical sample testing, D2 further demonstrated its potential for clinical application. For comparison, erythrocytes were treated with DTT following the ÖGBT protocol, which is comparable to that described in Judd’s Methods in Immunohematology (4th edition) but differs from the AABB standard procedure using 0.2 mol/L DTT at pH 8.0. Unexpectedly, residual agglutination was still observed in 10 samples after DTT treatment. Notably, four of these samples (from patients 1, 22, 25, and 42) were collected just 2 days after the last DARA administration, and one sample (from patient 44) was collected 4 days after administration. All these patients had received DARA or ISA therapy with suboptimal clinical response, and progressive disease was observed. Given that DARA exhibits non-linear pharmacokinetics, with decreased clearance at higher doses and over time due to target-mediated effects, the persistent weak agglutination observed in patients 1, 20, 22, 25, 40, 42, 44, 46, 47, and 48 after DTT-treated RBC testing may be explained by residual high levels of anti-CD38 mAb (37). These observations are consistent with findings reported by C.P. Habicht and C. Schneeweiss (29), supporting the notion that residual reactivity after DTT treatment can be sample-dependent and influenced by factors such as dosing interval, cumulative exposure, and therapeutic response. Importantly, in our study, D2-treated RBCs effectively eliminated anti-CD38 mAb interference in all patient samples. Although some agglutination remained in Cell II and Cell III, this was attributable to the presence of specific clinically relevant antibodies, as confirmed for patients 9 (IgG anti-Wr^a^) and 24 (IgG anti-E). These results indicate that D2 treatment is more effective than DTT in removing anti-CD38 interference while preserving the detection of irregular antibodies, particularly in samples with high antibody titers or collected shortly after therapy. In contrast, D2 treatment appears unaffected by these variables, making it a more robust and reliable approach for irregular antibody detection in patients receiving anti-CD38 therapy.

To further validate its clinical utility, clinically relevant irregular antibody samples (IgG anti-D, anti-C, anti-E, anti-Fy^b^, anti-Jk^a^, anti-Jk^b^, anti-Le^a^, anti-Di^a^, anti-Di^b^, and anti-S) and commercial antisera (IgG anti-K and anti-Fy^a^) were tested with D2-treated RBCs spiked with 0.5 mg/mL DARA or ISA. D2 consistently eliminated pan-agglutination caused by anti-CD38 mAbs while preserving reactivity with all irregular antibodies. All antibodies were reproducibly detected using the same sensitivity and agglutination strength as in samples without added DARA or ISA (**Figures 6A–L**; **Supplementary Figures S2A–L**). Low-titer antibodies, including IgG anti-E, anti-Fy^b^, anti-Jk^a^, anti-Jk^b^, and anti-S, maintained agglutination strength equivalent to controls after D2 treatment. For commercial antisera, testing was performed after serial dilution to approximate the detection limit, and D2 similarly did not influence antibody reactivity. These findings confirm that D2 effectively mitigates therapeutic anti-CD38 interference without compromising the detection of clinically significant irregular antibodies.

Although only two clinical samples from ISA-treated patients were available for this study, as ISA has recently been introduced in China, future work will expand sample inclusion to further confirm the broad applicability of D2 across different CD38-targeted therapies. D2-treated RBCs remained stable for at least 6 months at 4°C, demonstrating excellent storage stability and operational feasibility.

While D2 effectively masks currently used anti-CD38 antibodies, the potential introduction of novel CD38-targeted agents with distinct epitope profiles necessitates further evaluation of D2’s binding versatility. A combinatorial masking approach may ultimately be required for universal applicability. Nevertheless, the successful development and validation of D2 provides a model for addressing similar serologic interference from other therapeutic mAbs. Ongoing research in our group aims to extend this strategy to other antibody targets, such as CD47, whose high expression on RBCs suggests a similar potential to cause pan-agglutination in pretransfusion testing.

In conclusion, D2 is a stable, safe, and easy-to-use rabbit mAb that effectively eliminates anti-CD38 therapeutic antibody-induced interference in pretransfusion testing. By preserving antigen integrity and test sensitivity, D2 offers a novel solution to improve transfusion safety and diagnostic accuracy in patients receiving CD38-targeted therapies. Further large-scale and multicenter validation studies will be required to support widespread clinical adoption, but current evidence supports D2 as a promising and practical advance for modern transfusion medicine.

## Data Availability

The original contributions presented in the study are included in the article/**Supplementary Material**. Further inquiries can be directed to the corresponding author.

## Supplementary material

Supplementary Figure 1 Comparison of antigen detection in NC, D2-, and DTT-treated RBCs. **(A–B)** The antigen detection results of untreated RBCs, D2-treated RBCs, and DTT-treated RBCs were compared using panel cells. NC, untreated RBCs; D2, D2-treated RBCs; DTT, DTT-treated RBCs.

Supplementary Figure 2 D2-treated eliminates the interference caused by ISA without affecting the detection of irregular antibodies. Results of indirect antihuman globulin (Coombs’) tests of red-cell antibody-positive plasma specimens in the presence (plus sign) or absence (minus sign) of isatuximab and D2 are shown. Plasma or serum specimens obtained from patients with known red-cell irregular antibodies, or from commercial serum samples, were spiked with isatuximab to a final concentration of 0.5 mg/mL and then tested. **(A)** IAT results showing that D2-treated RBCs enabled detection of anti-D in plasma containing ISA. **(B)** or anti-C. **(C)** or anti-E. **(D)** or anti-Fy^a^. **(E)** or anti-Fy^b^. **(F)** or anti-Jk^a^. **(G)** or anti-Jk^b^. **(H)** or anti-Le^a^. **(I)** or anti-Di^b^. **(J)** or anti-S. **(K)** or anti-Di^a^. **(L)** Comparison of IAT results for anti-K detection using D2-treated versus DTT-treated RBCs in plasma containing ISA. A solid pellet at the bottom of the tubes indicates a negative result, and suspended particles (red-cell agglutinates) within the gel matrix indicate a positive test result (either a 1+ or 2+ degree of agglutination).
