# Supplementary figures and images for: A rabbit anti-human CD38 antibody for eliminating daratumumab and isatuximab interference in immunohematology testing
Source: Front Immunol. 2026 Feb 10;17:1726341. doi: 10.3389/fimmu.2026.1726341 (PMC12929416; doi:10.3389/fimmu.2026.1726341)

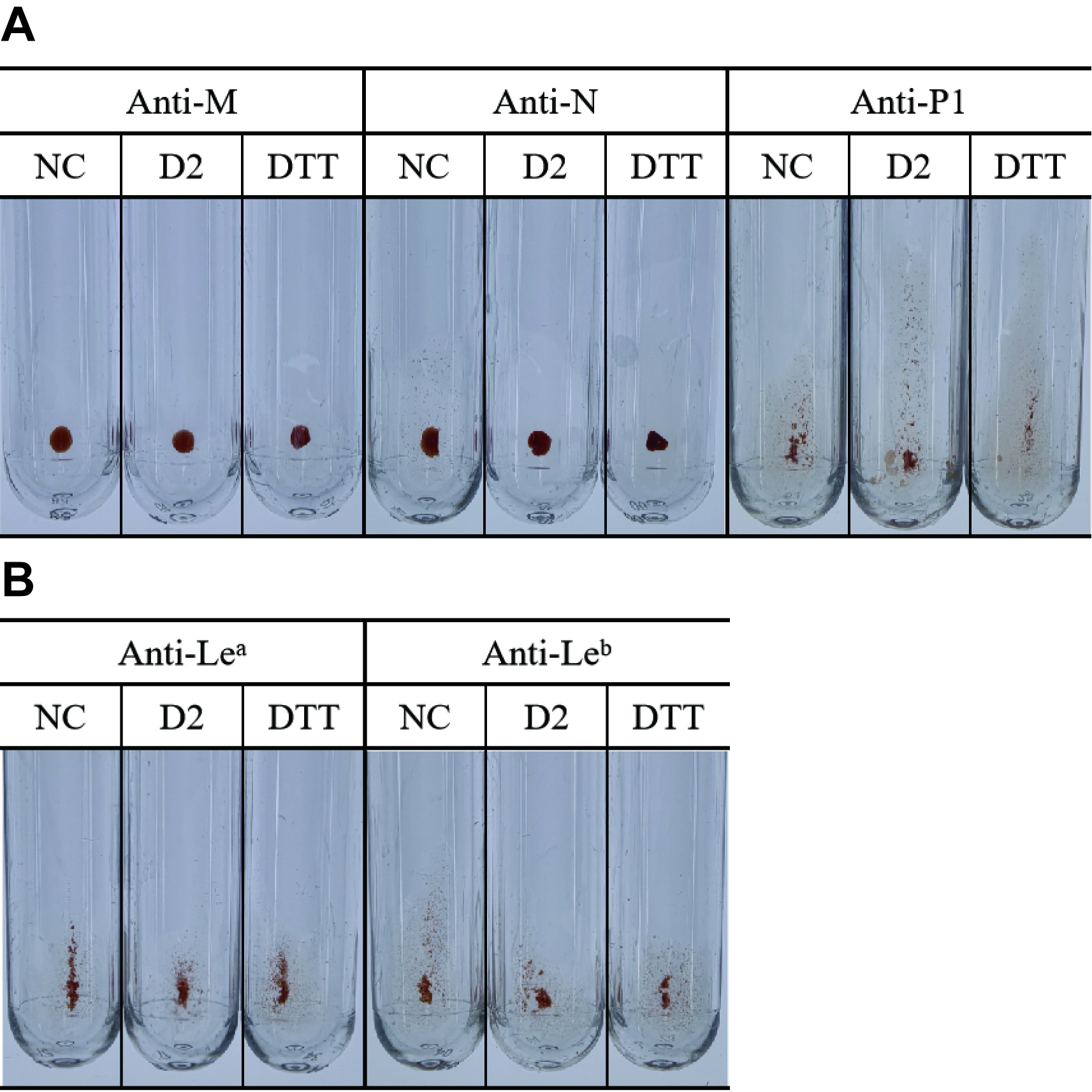

Supplement: Supplementary Figure 1 — Comparison of antigen detection in NC, D2-, and DTT-treated RBCs. (A–B) The antigen detection results of untreated RBCs, D2-treated RBCs, and DTT-treated RBCs were compared using panel cells. NC, untreated RBCs; D2, D2-treated RBCs; DTT, DTT-treated RBCs. [file Image1.tif]

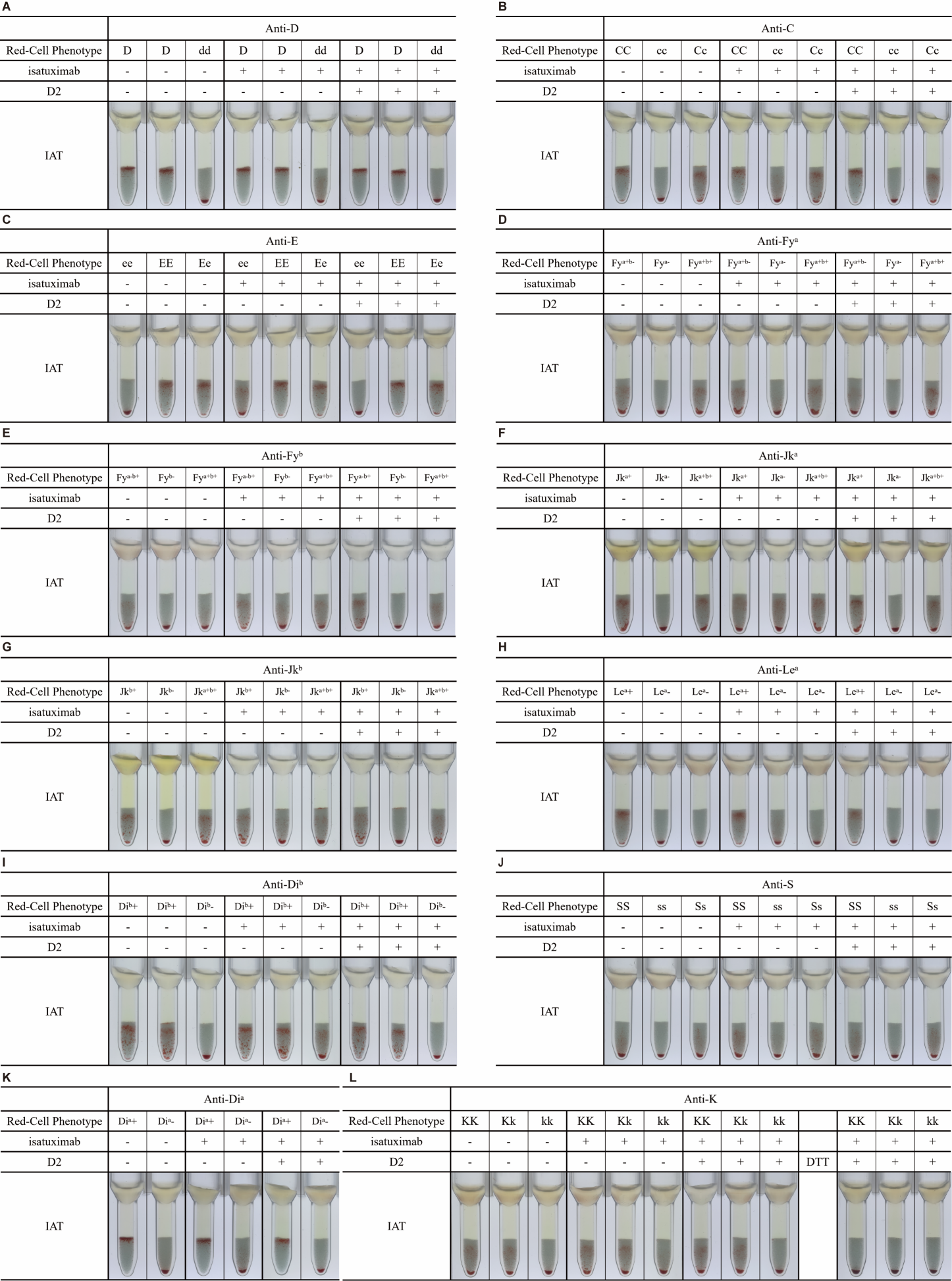

Supplement: Supplementary Figure 2 — D2-treated eliminates the interference caused by ISA without affecting the detection of irregular antibodies. Results of indirect antihuman globulin (Coombs’) tests of red-cell antibody-positive plasma specimens in the presence (plus sign) or absence (minus sign) of isatuximab and D2 are shown. Plasma or serum specimens obtained from patients with known red-cell irregular antibodies, or from commercial serum samples, were spiked with isatuximab to a final concentration of 0.5 mg/mL and then tested. (A) IAT results showing that D2-treated RBCs enabled detection of anti-D in plasma containing ISA. (B) or anti-C. (C) or anti-E. (D) or anti-Fya. (E) or anti-Fyb. (F) or anti-Jka. (G) or anti-Jkb. (H) or anti-Lea. (I) or anti-Dib. (J) or anti-S. (K) or anti-Dia. (L) Comparison of IAT results for anti-K detection using D2-treated versus DTT-treated RBCs in plasma containing ISA. A solid pellet at the bottom of the tubes indicates a negative result, and suspended particles (red-cell agglutinates) within the gel matrix indicate a positive test result (either a 1+ or 2+ degree of agglutination). [file Image2.tiff]
