# Supplementary material for: A rabbit anti-human CD38 antibody for eliminating daratumumab and isatuximab interference in immunohematology testing
Source: Front Immunol. 2026 Feb 10;17:1726341. doi: 10.3389/fimmu.2026.1726341 (PMC12929416; doi:10.3389/fimmu.2026.1726341)
Supplement: Supplementary file 3 [file DataSheet1.docx]

Table S1. Primers used for amplification of CD38 gene

| Gene（human） | Primer | Primer sequences（5’-3’） |
| --- | --- | --- |
| CD38 | F | CTGTGGCTGAGAGGTGCCAGATGTGTCCCGAGGTGGCGCCAGCAGTG |
|  | R | GAATTCATCAGTGGTGGTGGTGGTGGTGGCTGCCGCCGCCGCCGATCTCAGATGTGCAA |
