# Supplementary material for: A rabbit anti-human CD38 antibody for eliminating daratumumab and isatuximab interference in immunohematology testing
Source: Front Immunol. 2026 Feb 10;17:1726341. doi: 10.3389/fimmu.2026.1726341 (PMC12929416; doi:10.3389/fimmu.2026.1726341)
Supplement: Supplementary file 4 [file DataSheet2.docx]

Table S2. RT-PCR reaction program for rabbit single B cells

| Program | Temperature | Time | Cycles |
| --- | --- | --- | --- |
| Reverse transcription | 50℃ | 60 min | 1 |
| Primary PCR |  |  |  |
| Pre-denaturation | 95℃ | 3 min | 1 |
| Denaturation | 95℃ | 15 s | 20 |
| Annealing&Extension | 60℃ | 15 min |  |
| Nested PCR |  |  |  |
| Pre-denaturation | 98℃ | 3 min | 1 |
| Denaturation | 98℃ | 15 s | 35 |
| Annealing | 60℃ | 30 s |  |
| Extension | 72℃ | 15 s |  |
| Final hold | 4℃ | ∞ | 1 |
